# Morphology of the anterolateral ligament: a complex of fibrous tissues spread to the anterolateral aspect of the knee joint

**DOI:** 10.1007/s12565-020-00543-1

**Published:** 2020-04-28

**Authors:** Hisayo Nasu, Akimoto Nimura, Kumiko Yamaguchi, Keiichi Akita

**Affiliations:** 1 Department of Clinical Anatomy, Graduate School of Medical and Dental Science, Tokyo Medical and Dental University, 1-5-45 Yushima, Bunkyo-ku, Tokyo, 113-8519 Japan; 2 Department of Functional Joint Anatomy, Graduate School of Medical and Dental Science, Tokyo Medical and Dental University, Tokyo, Japan; 3 Professional Development in Health Sciences, Graduate School of Medical and Dental Science, Tokyo Medical and Dental University, Tokyo, Japan

**Keywords:** Anatomy, Anterolateral ligament, Knee joint, Sheet-like structure

## Abstract

The anterolateral ligament (ALL) has recently received considerable attention as a key structure maintaining the rotational stability of the tibia. However, the morphology of the ALL, particularly the proximal attachment, is controversial. This study aimed to elucidate the morphological relationship between the ALL and its adjacent structures. A total of 25 knees from 22 cadavers were used in the current study. One knee was set at 30°, 60° and 90° of flexion. Stretched or winkled fibrous tissues were then observed with internal and external rotations of the tibia at each angle. In 22 knees, fibrous tissues that were attached to the lateroposterior area to the Gerdy’s tubercle were macroscopically observed. In the other 2 knees, the fibrous tissues were histologically investigated and analyzed using computer-assisted three-dimensional reconstruction. A taut fibrous tissue was observed between the lateroposterior area to the Gerdy’s tubercle and the posterosuperior area to the lateral epicondyle during an internal rotation of the tibia. A complex of fibrous tissues that were attached to the lateroposterior area to the Gerdy’s tubercle spread to the anterolateral aspect of the knee as a sheet-like structure. This complex tissue was composed of the fascia lata and fibrous tissues continuous from the fabellofibular ligament, intermuscular septum, and tendon of the gastrocnemius. Three-dimensional reconstruction showed that each fibrous tissue formed a sheet. The structure recognized as the ALL could not be detected; therefore, the ALL that has been reported to date is considered to be a complex of fibrous tissues with a sheet-like structure.

## Introduction

A distinct ligamentous structure was rediscovered at the anterolateral site of the knee joint in 2013 (Claes et al. 2013). Since then, the anterolateral ligament (ALL) has been reported by numerous researchers because the ALL is regarded as one of the key structures to maintain the rotational stability of the tibia after anterior cruciate ligament (ACL) reconstruction. However, the morphology of the ALL has been controversial. Some researchers (Vincent et al. 2012; Claes et al. 2013; Helito et al. 2013; Macchi et al. 2016) reported that the ALL is a distinct ligamentous structure. On the other hand, De Maeseneer et al. (2015) found that the ALL does not always present as a thick, well-identifiable ligament, but more typically as a thinner structure with delicate fibers.

With regard to the attachments of the ALL, many researchers (Claes et al. 2013; Dodds et al. 2014; Porrino et al. 2015; De Maeseneer et al. 2015; Coquart et al. 2016; Caterine et al. 2017) reported that the distal attachment is located posterior to the Gerdy’s tubercle. However, descriptions on the proximal attachment are inconsistent. Dodds et al. (2014) demonstrated that the femoral attachment of the ALL is a mean of 8 mm proximal and 4.3 mm posterior to the most prominent point of the lateral epicondyle. On the other hand, Caterine et al. (2017) explained that the proximal attachment is not clearly visible due to the close relationship of the ALL with the other ligamentous structures. In our previous study conducted from a perspective of the joint capsule, we found that the width of the joint capsular attachment is wider at the posterior area to the Gerdy’s tubercle and the joint capsule is attached to this area via developed uncalcified fibrocartilages (Nasu et al. 2017). The area mentioned above is equivalent to the distal attachment area of the ALL. Following fibrous tissues that were attached to the lateroposterior area to the Gerdy’s tubercle proximally might make the proximal attachment of the ALL clear.

Therefore, the aim of the current study was to elucidate the morphological relationship between fibrous tissues that were attached to the lateroposterior area to the Gerdy’s tubercle and the adjacent structures. We hypothesized that fibrous tissues that were attached to the lateroposterior area to the Gerdy’s tubercle could extend proximally as a sheet-like structure having connections with adjacent structures. Elucidating the morphology of the ALL could advance knowledge of the pathology of internal rotational instability and broaden the possibility of more appropriate conservative treatment.

## Materials and methods

A total of 25 knees from 22 cadavers (15 males and 7 females) were used in the current study. All the cadavers used were donated to Tokyo Medical and Dental University. Knees with a flexion contracture and a previous surgery were excluded. The average age was 80 years, and the range was 42–94 years. Twenty-one cadavers were fixed using 8% formalin and preserved in 30% ethanol. One cadaver was fixed using the Thiel’s method (1992), in which the joint movements were maintained as smoothly and flexibly as observed in the fresh cadavers. All procedures were approved by the institutional review board of Tokyo Medical and Dental University (Approval No. M2018-243).

In one knee fixed using the Thiel’s method, skin and subcutaneous tissues were removed, and the fascia lata was exposed. The fascia lata was cut at the midline of the posterior surface of the thigh and peeled anteriorly. After cutting the connection between the fascia lata and the intermuscular septum, the fascia lata was reflected more anteriorly and the iliotibial tract was detached from the Gerdy’s tubercle. The biceps femoris was then removed. The knee was set at 30°, 60° and 90° of flexion using a non-digital goniometer, and the tibia was rotated internally and externally at each angle. The specimen was observed at the lateral view, and the findings were photographed.

In 22 knees fixed using 8% formalin, the same dissection methods were conducted and the biceps femoris was reflected inferiorly. Next, the fibrous tissues on the fibular collateral ligament were cut and opened. The fibular collateral ligament was exposed, and the inferior lateral genicular artery and vein were identified. The findings were drawn and photographed.

Two knees were investigated histologically to analyze the origins of the fibrous tissues that were attached to the lateroposterior area to the Gerdy’s tubercle. The two specimens were sliced horizontally at 5 mm from the Gerdy’s tubercle to the lateral epicondyle. A diamond-band pathology saw (EXAKT 312; EXAKT Advanced Technologies, Norderstedt, Germany) was used to slice them. After fixation in 8% formalin and decalcification in a solution containing aluminum chloride, hydrochloric acid, and formic acid, as described by Plank and Rychlo (1952), the slices were trimmed to small blocks. The blocks were embedded in paraffin and sectioned at 5 µm. Sections were analyzed with Masson’s trichrome staining.

In one of the two specimens, fibrous tissues spreading to the lateral side of the knee were analyzed using computer-assisted three-dimensional (3-D) reconstruction. All sections were scanned and traced in Adobe Photoshop software (CS6, Adobe, San Jose, USA). The iliotibial tract was traced in dark green, the fascia lata in green, the fabellofibular ligament in yellow, the biceps femoris in brown, a fibrous tissue from the intermuscular septum in pink, tendon of the gastrocnemius in orange, muscle of the gastrocnemius in purple, a fibrous tissue from the tendon of the gastrocnemius in reddish purple, the fibular collateral ligament in sky blue, bone in gray, arteries in red, and veins in blue. Section sequences were reconstructed using SRFII software (TRI/3D-SRFII-L, RATOC SYSTEM ENGINEERING, Tokyo, Japan).

## Results

### Differences in how fibrous tissues were stretched at internal and external rotations of the tibia

**Fig. 1:**
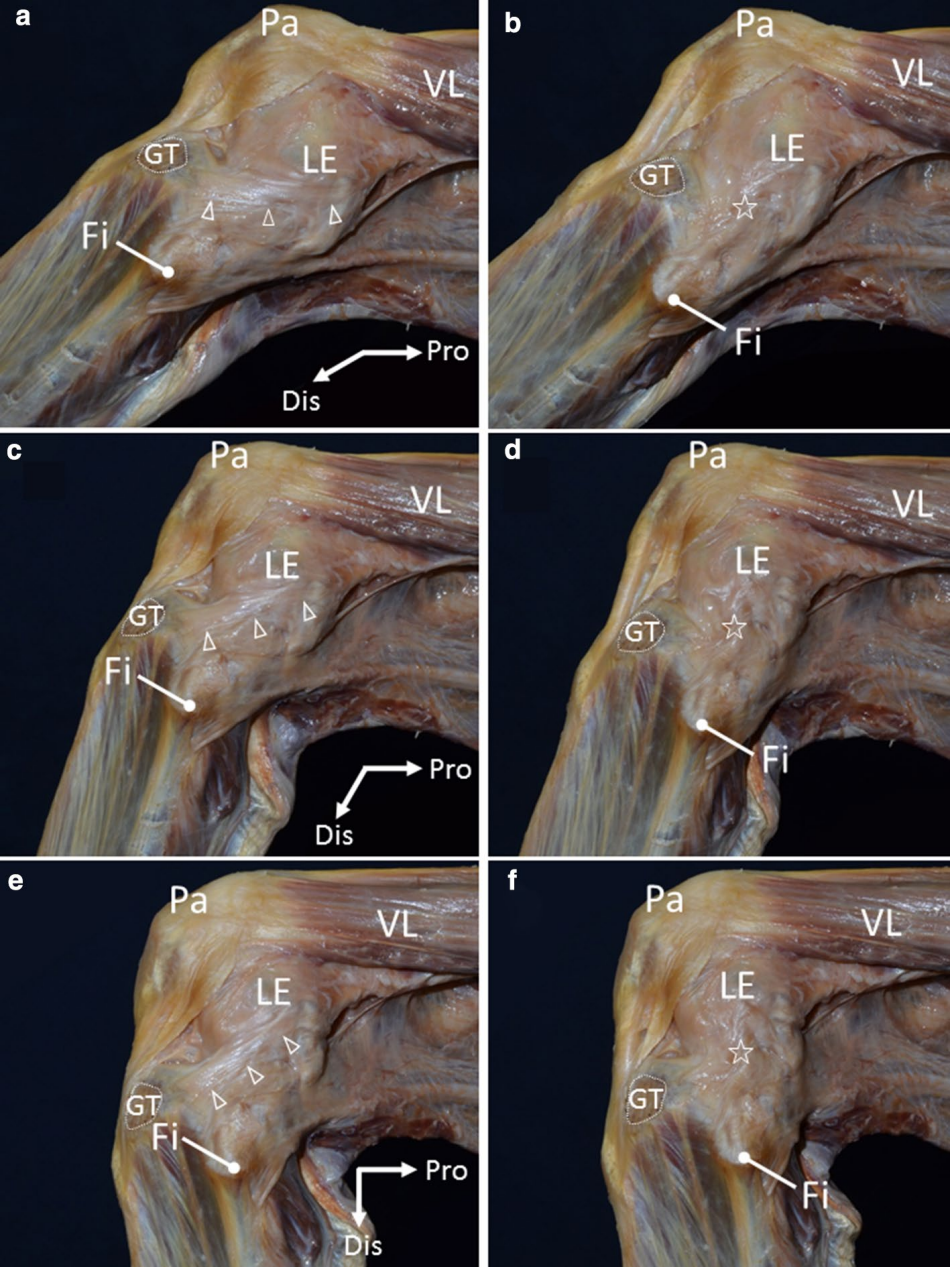
Differences in how fibrous tissues were stretched at internal and external rotations of the tibia. **A** Lateral view of the left knee at 30° of flexion with internal rotation of the tibia. White dotted line shows the Gerdy’s tubercle (GT). A taut fibrous tissue (arrowheads) was attached to the lateroposterior area to the GT, ascending toward the lateroposterior area of the lateral condyle of the femur, and connected to the posterosuperior area to the lateral epicondyle (LE). **b** Lateral view of the left knee at 30° of knee flexion with external rotation of the tibia. The taut fibrous tissue disappeared, and wrinkles of a sheet-like structure appeared (star). **c** At 60° of knee flexion with internal rotation of the tibia. **d** At 60° of knee flexion with external rotation of the tibia. **e** At 90° of knee flexion with internal rotation of the tibia. **f** At 90° of knee flexion with external rotation of the tibia. *Fi* fibula, *Pa* patella, *VL* vastus lateralis, *Dis* distal, *Pro* proximal

At 30° of knee flexion, the tibia was rotated internally and externally. At the internal rotation of the tibia, a taut fibrous tissue was observed on the lateral aspect of the knee. The taut fibrous tissue was attached to the lateroposterior area to the Gerdy’s tubercle, ascending toward the lateroposterior area of the lateral condyle of the femur, and connected to the posterosuperior area to the lateral epicondyle (Fig. 1). On the other hand, at the external rotation of the tibia, the taut fibrous tissue disappeared and wrinkles of a sheet-like structure appeared (Fig. 1).

At 60 and 90° of knee flexion, fibrous tissues on the lateral side of the knee appeared like a cord structure connecting the lateroposterior area to the Gerdy’s tubercle and posterosuperior area to the lateral epicondyle with internal rotation of the tibia. However, the cord-like structure was not observed with external rotation of the tibia.

### Structures surrounding the lateral side of the knee

**Fig. 2:**
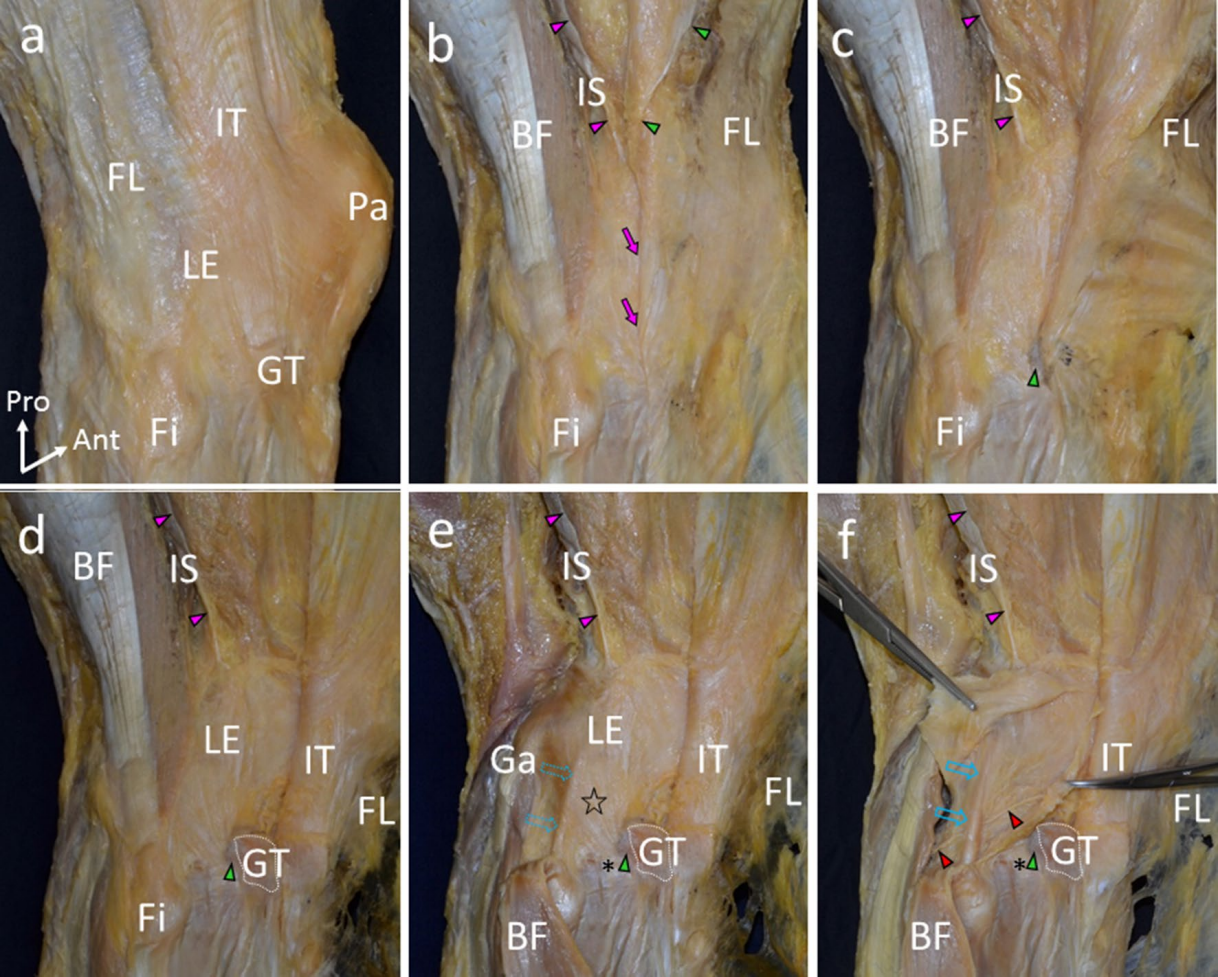
Structures surrounding the lateral side of the knee. **a** Posterolateral aspect of the right knee. The iliotibial tract (IT) was attached to the Gerdy’s tubercle (GT). **b** The fascia lata (FL) was reflected anteriorly. A connection between the intermuscular septum (IS) and the FL was cut (pink and green arrowheads). A fibrous tissue continuous from the IS was mingled with the deep surface of the FL (pink arrows). **c** The FL was reflected anteriorly more. A part of the FL was attached lateroposterior to the GT (Green arrowhead). **d** The IT was detached from the GT and reflected anteriorly. **e** The biceps femoris (BF) was reflected inferiorly. Fibrous tissues were attached to the lateroposterior area (asterisk) to the GT. The fibrous tissues extended to the posterosuperior area of the lateral condyle. They were observed as a sheet (star) on the fibular collateral ligament (dotted arrows in sky blue). f. After cutting and opening the sheet, the fibular collateral ligament (arrows in sky blue) was observed. The inferior lateral genicular artery (red arrowheads) and vein ran deep to the fibular collateral ligament and went forward under the sheet. *Fi* fibula, *Ga* gastrocnemius, *LE* lateral epicondyle, *Pa* patella, *Ant* anterior, *Pro*, proximal

After reflecting the fascia lata anteriorly, a connection between the fascia lata and the intermuscular septum was observed (Fig. 2). While cutting the connection from proximal to distal, the distal part of the intermuscular septum became a tendinous structure and was attached proximal to the lateral epicondyle. A part of the intermuscular septum descended as a fibrous tissue distal to the lateral condyle without an attachment to the proximal area to the lateral epicondyle. It was observed under the biceps femoris and was connected with the deep surface of the fascia lata (Fig. 2).

A part of the fascia lata was attached lateroposterior to the Gerdy’s tubercle, and the iliotibial tract was inserted in the Gerdy’s tubercle. However, a boundary between the fascia lata and the iliotibial tract was not clear. After each of them was detached from the tibia and the biceps femoris was reflected inferiorly, fibrous tissues that were attached to the lateroposterior area to the Gerdy’s tubercle were exposed (Fig. 2). The fibrous tissues spread to the posterosuperior area of the lateral condyle as a sheet-like structure. The fibular collateral ligament was positioned beneath the fibrous tissues. In addition, the inferior lateral genicular artery and vein ran deep to the fibular collateral ligament and went forward under the fibrous tissues (Fig. 2). However, any cord-like structures could not be observed beneath the fibrous tissues in all specimens.

### Fibrous tissue continuous from the fabellofibular ligament, intermuscular septum, and the tendon of the gastrocnemius

**Fig. 3:**
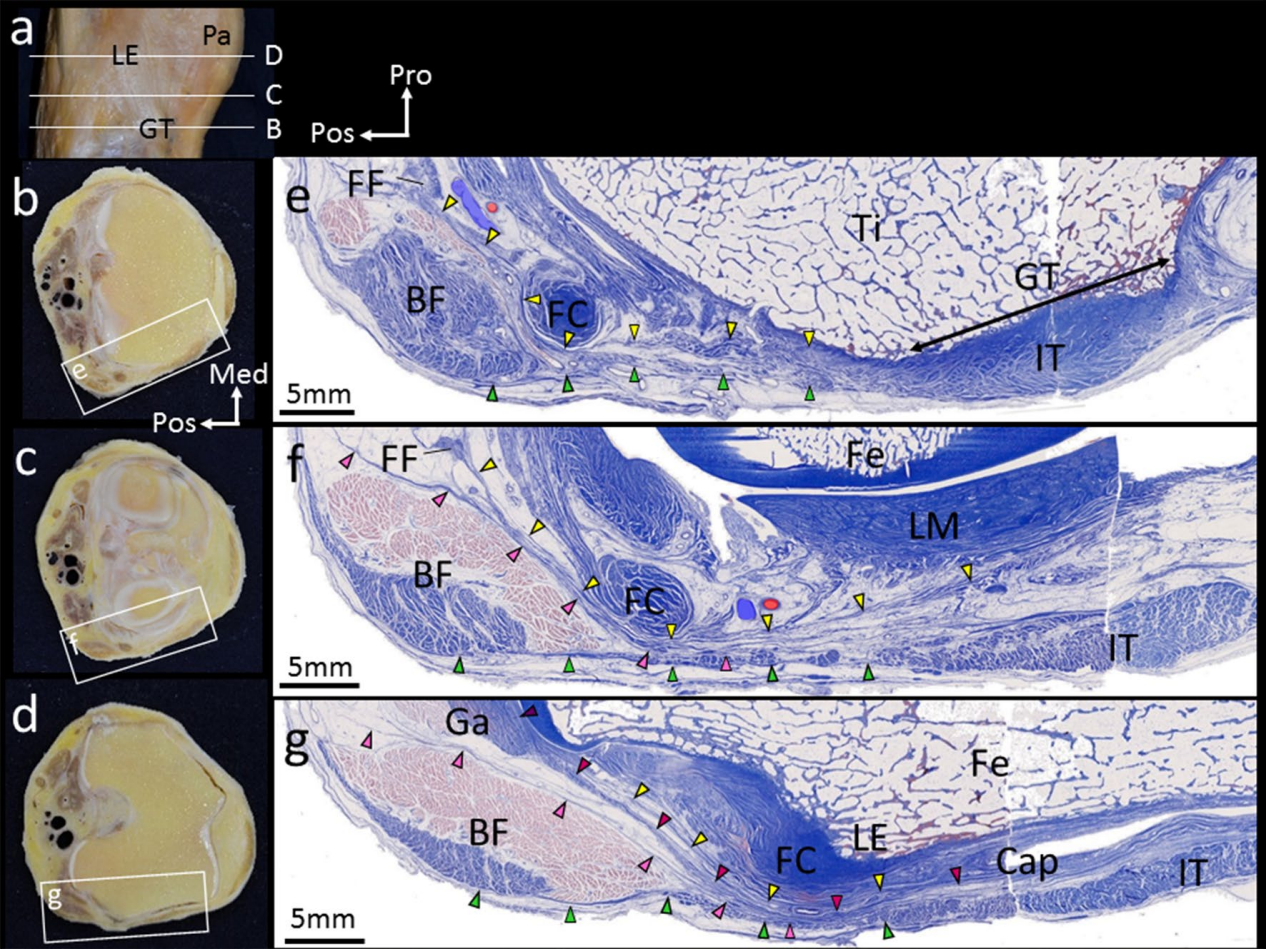
Fibrous tissue continuous from some structures. **a** Lateral aspect of the right knee. **b**–**d** Horizontal slices at lines (B), (C), and (D) in **a**. However, these slices are different from the sample of **a**. **e**–**g** Sections stained with Masson’s trichrome on rectangles in **b**, **c**, and **d**. **e** The range of the Gerdy’s tubercle (GT) is shown by a double arrow. The fascia lata (green arrowheads) and a fibrous tissue (yellow arrowheads) continuous from the fabellofibular ligament (FF) were attached to the lateroposterior area to the GT. **f** A fibrous tissue (pink arrowheads) was observed under the biceps femoris (BF). It was mingled with the fascia lata (green arrowheads). The fibrous tissue (yellow arrowheads) continuous from the FF adhered to the joint capsule or the lateral meniscus (LM). **g** A fibrous tissue (reddish purple arrowheads) continuous from the tendon (purple arrowhead) of the gastrocnemius (Ga) was observed. It was mingled with the joint capsule (Cap). *FC* fibular collateral ligament, *Fe* femur, *IT* iliotibial tract, *LE* lateral epicondyle, *Pa* patella, *Ti* tibia, *Med* medial, *Pos* posterior, *Pro* proximal

Sections at the Gerdy’s tubercle level showed two fibrous tissues gathering at the lateroposterior area to the Gerdy’s tubercle: one was the fascia lata and the other was a fibrous tissue continuous from the fabellofibular ligament (Fig. 3). Sections at the lateral meniscus level showed the fibrous tissue continuous from the fabellofibular ligament adhered to the joint capsule or the lateral meniscus. In addition to the fibrous tissue continuous from the fabellofibular ligament, a fibrous tissue was observed under the biceps femoris. Based on macroscopic findings, it was identified as a fibrous tissue continuous from the intermuscular septum. The fibrous tissue continuous from the intermuscular septum was combined with the deep surface of the fascia lata (Fig. 3). Sections at the lateral epicondyle level showed that a fibrous tissue was continuous from the tendon of the gastrocnemius and covered the joint capsule (Fig. 3). These fibrous tissues were located superficial to the fibular collateral ligament and the inferior lateral genicular artery and vein.

**Fig. 4:**
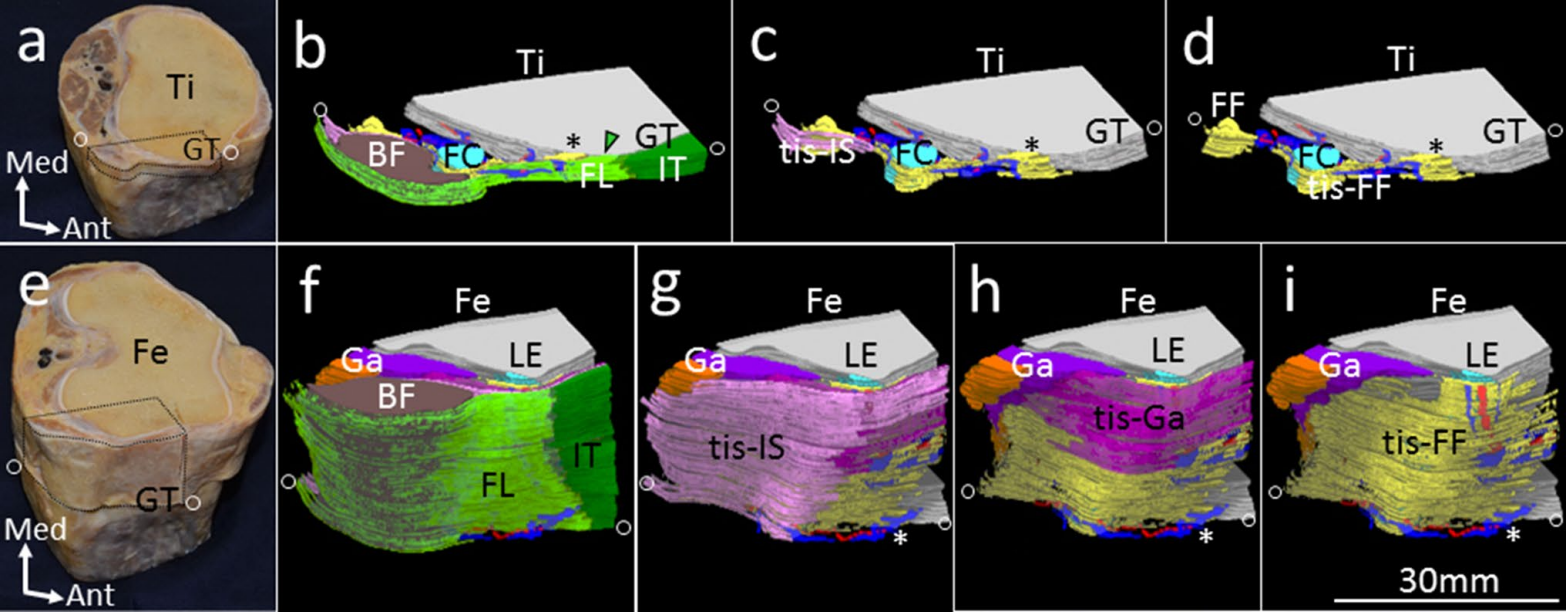
Three-dimensional reconstruction of fibrous tissues spread to the lateral side of the knee. **a** Superior aspect of the right tibia viewed from the posterolateral side. **b**–**d** Three-dimensional reconstruction built in a dotted area in **a**. However, the sample which the 3-D reconstruction is conducted is different from the specimen in **a**. Position of circles corresponds to their level in **a**. **b** The fibrous tissues attached to the lateroposterior area to the Gerdy’s tubercle (GT) are shown in green (green arrowhead) and yellow (asterisk). Green indicates the fascia lata (FL). **c** An image removing the FL, iliotibial tract (IT), and biceps femoris (BF). A fibrous tissue (tis-IS) continuous from the intermuscular septum shown in pink was placed under the BF. **d** An image removing the tis-IS. Yellow indicates a fibrous tissue (tis-FF) continuous from the fabellofibular ligament (FF). **e** Specimen adding more proximal mass of **a**. Superior aspect of the right femur viewed from the posterolateral side. **f**–**i** 3-D reconstruction built in a dotted area in **e**. Position of circles corresponds to their level in **e**. **f** The FL and IT surrounded the knee joint. **g** An image removing the FL, IT, and BF. The fibrous tissue (tis-IS) continuous from the intermuscular septum shown in pink extended toward the lateral side of the knee. Asterisk shows the area where the tis-FF is attached lateroposterior to the Gerdy’s tubercle. **h** An image removing the tis-IS. A fibrous tissue (tis-Ga) continuous from the tendon of the gastrocnemius (Ga) shown in reddish purple extended to the lateral side of the knee. **i** An image removing the tis-Ga. The fibrous tissue (tis-FF) continuous from the FF shown in yellow extended on the lateral aspect of the knee. *FC* fibular collateral ligament, *Fe* femur, *LE* lateral epicondyle, *Ti* tibia, *Ant* anterior, *Med* medial

By reconstructing these sections (Fig. 4), the fibrous tissue continuous from the fabellofibular ligament indicated in yellow extended to the lateral side of the knee as a sheet-like structure. On its superficial layer, the fibrous tissue continuous from the tendon of the gastrocnemius was composed of a sheet indicated in reddish purple. Additionally, on its more superficial layer, the fibrous tissue continuous from the intermuscular septum formed a sheet indicated in pink. The outermost layer was composed of the fascia lata indicated in green and the iliotibial tract indicated in dark green (Fig. 4). None of the cord-like structures on the superficial layer of the fibular collateral ligament were reconstructed.

## Discussion

The anterolateral ligament has been identified as a distinct ligamentous structure connecting the proximal tibia posterior to the Gerdy’s tubercle and the lateral epicondyle and/or its vicinity (Vincent et al. 2012; Claes et al. 2013; Helito et al. 2013; Dodds et al. 2014; Coquart et al. 2016; Macchi et al. 2016). However, we could not detect a distinct cord-like structure connecting these two points as expected to be located superficial to the fibular collateral ligament. Alternatively, we found that a complex of fibrous tissues spread to the anterolateral aspect of the knee joint as a sheet-like structure. The complex tissue was composed of the fascia lata, a fibrous tissue continuous from the fabellofibular ligament, a fibrous tissue continuous from the tendon of the gastrocnemius, and a fibrous tissue continuous from the intermuscular septum.

### Actual morphology of the structure recognized as the ALL

At tibial internal rotation, a taut fibrous tissue between the lateroposterior area to the Gerdy’s tubercle and the posterosuperior area to the lateral epicondyle appeared. However, the taut fibrous tissue disappeared, and wrinkles of a sheet-like structure appeared at external rotation of the tibia. This result is consistent with a description that a pearly fibrous band is exaggerated with extreme tension at internal rotation by Segond (1879). O’Driscoll et al. (1992) also described the same phenomenon about the lateral ulnar collateral ligament in the elbow joint. They explained that the lateral ulnar collateral ligament is a capsulo-ligamentous complex that stands out when varus stress is applied. We also consider that the ALL, which has attracted considerable attention, could be a complex of fibrous tissues that stand out when the tibia is rotated internally. The term “ligament” has broad definitions. A ligament includes not only a structure connecting bone to bone, but also an aponeurosis-like structure or a tendon-like structure (Schleip et al. 2012). We suggest that the structure recognized as the ALL should be defined as a complex of fibrous tissues closer to an aponeurosis.

### Extension of fibrous tissues attaching to the lateroposterior area to the Gerdy’s tubercle

As a result of following the fibrous tissues that were attached to the lateroposterior area to the Gerdy’s tubercle, the fibrous tissues were composed of two structures: one was the fascia lata and the other was a fibrous tissue continuous from the fabellofibular ligament.

The fascia lata stated above is regarded to be synonymous to the posterior fibers of the iliotibial tract because the iliotibial tract is a thick part of the fascia lata and their boundary of them cannot be identified. Milch et al. (1936) mentioned that an avulsion is caused by tension on the iliotibial tract at its insertion into the area behind the Gerdy’s tubercle during the internal rotation of the tibia with the knee in the partly flexed position. We consider that the fascia lata, attached to the lateroposterior area to the Gerdy’s tubercle, could be a key fiber concerned with pathologies of internal rotational stability and avulsion fracture.

We also found that the fibrous tissue continuous from the fabellofibular ligament contributed to the attachment to the lateroposterior area to the Gerdy’s tubercle. The fabellofibular ligament was not recognized in all cadavers but present in 42.1% (Kim et al. 1997). The thickness of the fibrous tissue might vary depending on the development of the fabellofibular ligament. The fibrous tissue continuous from the fabellofibular ligament gradually tapered toward the proximal area of the lateral condyle, and the fibrous tissue continuous from the tendon of the gastrocnemius spread to the more proximal area. This extension of fibrous tissues could be attributed to the variations on the width of the proximal attachment of the ALL: 11.85 mm by Daggett et al. (2016), 8.3 mm by Claes et al. (2013), and 4.8 mm by Caterine et al. (2017). Defining the proximal attachment of the ALL as a point could be difficult.

### Terminologies of structures supporting the anterolateral aspect

The ALL is considered to be the same as what had been previously referred to as lateral capsular ligament or capsulo-osseous layer of the iliotibial tract (Macchi et al. 2016). The lateral capsular ligament or capsulo-osseous layer has a connection with the fascia covering the gastrocnemius (Johnson 1979; Terry et al. 1986). In addition, Herbst (2017) described that the capsulo-osseous layer was continuous with the fascia of not only the gastrocnemius, but also the biceps femoris. From our results, based on 3-D reconstruction, we consider that the fibrous tissues continuous from the tendon of the gastrocnemius and fabellofibular ligament, and intermuscular septum under the biceps femoris were combined and formed the capsulo-osseous layer described previously.

### Clinical significance of a complex of fibrous tissues in the lateral side of the knee

Sonnery-Cottet et al. (2015) described that a combined ACL and ALL reconstruction allows good anteroposterior and rotational laxity control without specific complications, such as stiffness and limited range of motion. By contrast, Schon et al. (2016) demonstrated that an ALL reconstruction at any flexion angle overconstrains native joint kinematics. Herbst et al. (2018) proved that the lateral extra-articular tenodesis with ACL reconstruction overconstrains the tibial rotational range when it is performed in knees with an intact anterolateral capsule. Moreover, Guenther et al. (2017) focused on the anterolateral capsule, but not the ALL, and found that the anterolateral capsule transmits force between adjacent regions of the capsule rather than a longitudinal axis along with a ligament. They mentioned that the anterolateral capsule should be handled as a sheet of tissue rather than a composition of individual components. We totally agree the description of Guenther et al. based on our results in the current study.

Additionally, the gastrocnemius or biceps femoris could contribute to the tibial rotational stabilization via the complex of the fibrous tissues. Shultz et al. (2000) demonstrated that the gastrocnemius and the hamstring fire significantly faster than the quadriceps as recorded in the surface electromyography during either internal or external rotational moment of the trunk and femur relative to the weight-bearing tibia. Understanding the composition of this complex of fibrous tissues is useful for physical therapists because they have to treat joints considering that one structure could be affected by other structures. Our viewpoint that the structure recognized as the ALL is a complex of fibrous tissues could be significant to broaden their treatment strategy against internal rotational instability.

### Limitations

This study has three limitations. First, the age of the materials was imbalanced because most of the cadavers were from the elderly people. Second, the specimens were mixed with the use of both sides and only use of one side. Third, this was purely an anatomical study and did not include biomechanical or kinematic examinations.

## Conclusions

The structure recognized as the ALL could not be detected. We consider that the ALL, which has attracted considerable attention, may be a complex of fibrous tissues that stand out when the tibia is rotated internally. The actual morphology of the structure recognized as the ALL should be defined as a complex of fibrous tissues closer to the aponeurosis rather than a fibrous band connecting bone to bone.
